# HHMMiR: efficient *de novo *prediction of microRNAs using hierarchical hidden Markov models

**DOI:** 10.1186/1471-2105-10-S1-S35

**Published:** 2009-01-30

**Authors:** Sabah Kadri, Veronica Hinman, Panayiotis V Benos

**Affiliations:** 1 Lane Center for Computational Biology, Carnegie Mellon University, Pittsburgh, PA 15213, USA; 2 Department of Biological Sciences, Carnegie Mellon University, Pittsburgh, PA 15213, USA; 3 Department of Computational Biology, University of Pittsburgh, Pittsburgh, PA 15260, USA

## Abstract

**Background:**

*MicroRNA*s (miRNAs) are small non-coding single-stranded RNAs (20–23 nts) that are known to act as post-transcriptional and translational regulators of gene expression. Although, they were initially overlooked, their role in many important biological processes, such as development, cell differentiation, and cancer has been established in recent times. In spite of their biological significance, the identification of miRNA genes in newly sequenced organisms is still based, to a large degree, on extensive use of evolutionary conservation, which is not always available.

**Results:**

We have developed HHMMiR, a novel approach for *de novo *miRNA hairpin prediction in the absence of evolutionary conservation. Our method implements a *Hierarchical Hidden Markov Model *(HHMM) that utilizes region-based structural as well as sequence information of miRNA precursors. We first established a template for the structure of a typical miRNA hairpin by summarizing data from publicly available databases. We then used this template to develop the HHMM topology.

**Conclusion:**

Our algorithm achieved average sensitivity of 84% and specificity of 88%, on 10-fold cross-validation of human miRNA precursor data. We also show that this model, trained on human sequences, works well on hairpins from other vertebrate as well as invertebrate species. Furthermore, the human trained model was able to correctly classify ~97% of plant miRNA precursors. The success of this approach in such a diverse set of species indicates that sequence conservation is not necessary for miRNA prediction. This may lead to efficient prediction of miRNA genes in virtually any organism.

## Background

### MicroRNAs

**Figure 1:**
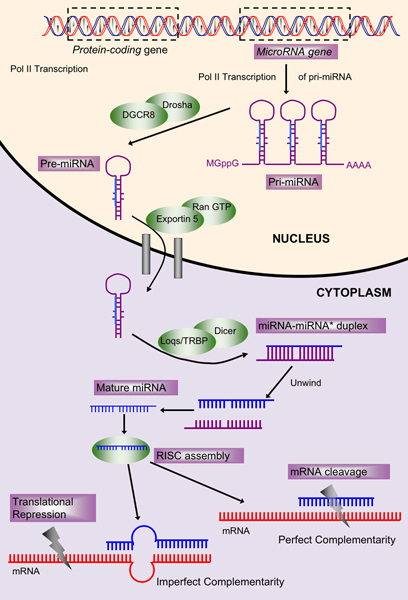
**Biogenesis of microRNAs**. miRNA genes are transcribed in the nucleus, where they undergo processing by DGCR8/Pasha and the RNAse III family enzyme, Drosha. The pre-miRNA is then transported into the cytoplasm where it is processed by Dicer, and the cofactor TRBP to generate a ~22 nt miRNA:miRNA* duplex. After unwinding, the miRNA forms part of the RISC assembly and causes mRNA degradation or translational repression.

*MicroRNA*s (miRNAs) are small (~22 nucleotide long) non-coding RNAs that are part of a eukaryote-specific system of gene regulation at the RNA level. MiRNAs act as post-transcriptional regulators of gene expression by base pairing with their target mRNAs. MiRNAs are primarily transcribed by *RNA Pol II *[1] as regions of longer RNA molecules (pri-miRNA) [2]. Individual pre-miRNA loops (~70 nts) are cleaved from the pri-miRNA by RNAse III enzyme, *Drosha *and transported into the cytoplasm by *RAN-GTP *and *Exportin 5 *[3] to be processed further to a ~22 nt long duplex, with 3' overhangs, by *Dicer *[4,5]. This duplex is commonly referred to as the miRNA:miRNA* duplex, where miRNA* is complementary to the miRNA. The miRNA:miRNA* duplex is subsequently unwound and the mature miRNA is loaded into multi-protein RISC (RNA-induced silencing complex) [6] while miRNA* usually degrades. In some cases, both miRNA and miRNA* are functional [7]. The miRNA biogenesis is illustrated in Figure 1. Mature miRNAs can cause translation inhibition or mRNA cleavage, depending on the degree of complementarity between the miRNA and its target sequence. Each miRNA can have multiple targets and each gene can be targeted by multiple miRNAs. It has been predicted that more than one third of human genes is regulated by miRNAs [8].

Plant and animal miRNAs differ not only in their biogenesis, but also in target-miRNA interactions. Plant miRNAs base pair with their targets with perfect or near-perfect complementarity and they regulate their targets mostly through mRNA cleavage at single sites in coding regions. Animal miRNAs usually base pair with 3' UTRs of the mRNAs at multiple target sites through imperfect complementarity. Due to these and other differences, it has been suggested that this regulation mechanism may have evolved independently in plants and animals [9]. Some viruses have also been shown to encode miRNAs that play a role in expression regulation of host genes [10].

### MiRNA identification

The first animal miRNA genes, *let-7 *and *lin-4*, were discovered in *Caenorhabditis elegans *by forward genetics [11-13]. Currently, miRNA genes are biochemically identified by cloning and sequencing size-fractionated cDNA libraries. The main limitation of this method is that lowly expressed miRNAs may be missed [14]. Although deep sequencing can help overcome this problem, this is currently a costly solution. Still, some miRNAs may be difficult to clone due to their sequence composition and possible post-transcriptional modifications [14-16]. Deep sequencing is being used on a large scale to identify small non-coding RNAs, but this is an expensive method and can only identify miRNAs expressed in a single cell type or in a given condition.

Computational predictive methods are fast and inexpensive and a number of approaches have been developed to predict miRNA genes, genome-wide. However, most of these approaches depend heavily on conservation of hairpins in closely related species [17-20]. Some methods have used clustering or profiling to identify miRNAs, [17,21,22]. The approach of Bentwich *et al. *[23] is interesting in that the whole genome is folded and scores are assigned to hairpins based on various features, including hairpin structural features and folding stability.

Machine learning approaches in the past have used support vector machines with high dimensional basis functions for classification of genomics hairpins [22,24,25]. Some of these methods depend on cross-species conservation for classification, while others do motif finding using multiple alignments. More recently, HMMs have been used in modelling miRNAs using both, evolutionary information and features related to the secondary structure [26].

### Hierarchical Hidden Markov Models

*Hierarchical Hidden Markov Models *(HHMMs) constitute a generalization of Hidden Markov Models (HMMs). They have been successfully used for modelling stochastic levels and length scales [27]. In biology, HHMMs have been used in the past to model vertebrate splice sites [28] and more recently in modelling *cis*-regulatory modules [29]. An HHMM has two types of states: *internal states *and *production states*. Each internal state has its own HHMM but cannot emit symbols by itself. It can activate a sub-state by a vertical transition. Sub-states can also make vertical transitions, until the lowest level in the hierarchy (production state) is reached. Production states are the only states that can emit symbols from the alphabet *via *their own probability distributions. Sub-states at the same level of hierarchy will be activated through horizontal transitions till an "end state" is reached. Every level has only one "end state" for each parent state that shifts control back to the parent. Thus, each internal state can emit sequences instead of single symbols. The node at the highest level of the hierarchy is called the "root" node while the leaf nodes are the productions states. Please refer to *Methods *for information about HHMM parameters and their estimation.

In this article, we report the results on the performance of an HHMM we developed for modelling miRNA hairpins. Although the model was trained on human sequences only, it was able to classify accurately hairpins from species as distant as worm, flies and plants, indicating that the degree of sequence and structural conservation for these genes may be high.

## Results

### Data summarization

**Figure 2:**
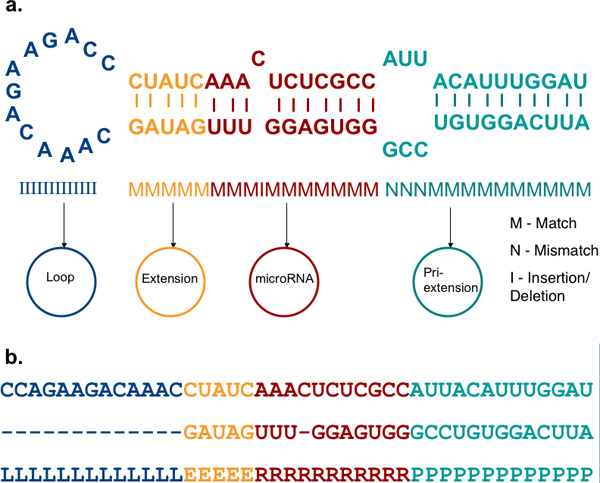
**The miRNA hairpin**. **(a) ***Template*: In our model, the miRNA precursor has four regions- "Loop" is the bulge and the *loop *state outputs *indels *only; "Extension" is a variable length region between the miRNA duplex and the loop; "microRNA" represents the duplex, without 3' overhangs; "Pri-extension" is the rest of the hairpin. The latter three states can output *matches*, *mismatches *and *indels*. (The nucleotides distribution and lengths are not to scale) **(b) ***Labelled precursor*: The precursor shown in (a) is labelled according to the regions it represents. This is the input format of training data for HHMMiR. L: Loop; E: Extension; R: MiRNA; P: Pri-miRNA.

We consider the hairpin stem-loop for predictions since it is structurally, the most prominent feature during biogenesis (Figure 1). MiRNA genes can be divided into four regions depicted in Figure 2a. After transcription, the RNA strand folds to form the hairpin precursor (Figure 1 and Figure 2a). The "loop" is the bulged end of the hairpin. The "miRNA" region defines the miRNA-miRNA* duplex (sans the 3' overhangs) that is processed by Dicer and further unwound. The region of the precursor extending from the end of the loop to the "miRNA" region is called the "extension". This region can be of variable length. The part of the hairpin sequence beyond the "miRNA" region may be part of the pri-miRNA in the nucleus and processed by Drosha. Thus, it has been named as "pri-extension", as suggested in Saetrom *et al. *[30].

The results presented in Table 1 show that the differences that exist between vertebrate and invertebrate miRNA genes are rather small. So, a probabilistic method trained in data from one organism is likely to perform well in another organism. As evident from the results in Table 1, the differences between length distributions of plant and animal precursors are relatively drastic, with the former having longer extension regions. The lengths of miRNAs and loops, however, are conserved across the two kingdoms. More information about species-specific differences is provided in Additional File 1. These genomes constitute an excellent test set for our algorithm in that they span various taxonomic groups, with different miRNA characteristics. Thus, it will be very useful to see how well an HHMM trained on (say) human sequences will be able to predict miRNA stem-loops in another vertebrate or invertebrate species and plants.

**Table 1:** Characteristics of miRNA hairpins in various taxonomic groups.

	**HP**	**LP**	**MIR**	**EXT**	**PRI**
**Mean**					
Vertebrates	86.7	7.3	22.0	5.0	12.6
Invertebrates	91.8	7.9	22.2	5.8	13.8
Plants	119.5	6.8	21.3	22.8	12.5
**Std. Dev**.					
Vertebrates	13.8	3.5	0.9	3.4	7.0
Invertebrates	13.1	3.9	1.3	4.5	5.9
Plants	43.2	3.7	1.0	18.5	9.9
**Minimum**					
Vertebrates	55	3	16	0	0
Invertebrates	54	3	18	0	0
Plants	57	3	16	0	0
**Maximum**					
Vertebrates	153	22	26	34	50
Invertebrates	215	30	28	55	32
Plants	337	35	24	102	78

*HP*: Hairpin length; *LP*: Loop length; *MIR*: MiRNA length; *EXT*: Distance of miRNA duplex from end of loop; *PRI*: Length of extension from end of miRNA to end of precursor. The list of organisms used for this Table is provided as *Supplementary Data*.

### HHMM model

HHMMiR is built around the miRNA precursor template illustrated in Figure 2a. The figure presents the four characteristic regions of stem-loop of a typical miRNA gene as described above. The length distributions of each of these regions are derived from Table 1. Each region, except the loop itself has three states: *match*, *mismatch*, and *insertion/deletion *(*indel*). *Match *means a base pairing at that position in the stem-loop, while *mismatch *means bulges on both arms at that position in the folded hairpin.*Indel *means that a base in one strand has no counterpart in the opposite strand. The loop will only have the *indel *state. Examples of these states are presented in Figure 2a.

**Figure 3:**
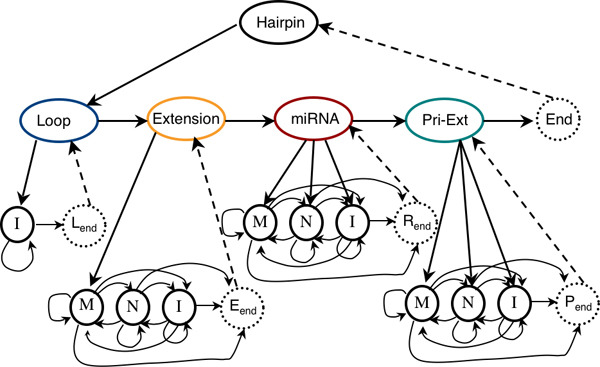
**The HHMM state model (based on the microRNA hairpin template)**. The oval shaped nodes represent the *internal states*. The colours correspond to the biological region presented in Figure 2a. The circular solid lined nodes correspond to the production states. The dotted lined states correspond to the silent end states. M: *Match *states, N: *Mismatch *states, I: *Indel *states, L_end_: Loop end state, R_end_: miRNA end state, P_end_: pri-extension end state.

The HHMM resulting from this scheme has three levels (Figure 3). *Hairpin *is the root node and can vertically transition to its *Loop *substate only. In our model, every hairpin begins with a loop. The four internal states at the second level correspond to the four main regions of the hairpin from Figure 2a. This level also has an *End *(L_end_) state to transfer control back to the *Hairpin*. Each internal state has a probabilistic model at the next lower level. A *Loop *cannot have base pairs and thus, has only one substate: *I *(*Indel). *The *Extension *state can only emit an *M *(*match*) state, when entered, since a mismatch or indel would become part of the loop. The *miRNA *and *pri-Ext *states can begin with a match, mismatch or indel. Each of these states has an *End *state (L_end_, R_end_, P_end _respectively)(see Figure 3).

### Datasets and alphabet selection

The training dataset contained a total 527 human miRNA precursors (positive dataset) and ~500 random hairpins (negative dataset), based on criteria derived from summarization (see *Methods*). The *RNAfold *program from Vienna Package [31] was used to obtain the secondary structure of these hairpins with the *minimum fold energy *(*mfe*). The parameters of the model were estimated using a modified Baum-Welch algorithm (see *Methods *for details on data sets and algorithms). All tests were conducted with 10-fold cross validation with random sampling.

We tested our model on two alphabets: Σ_1 _with *matches M *= {AU, GC, GU}, *indels I *= {A-, G-, C-, U-} and *mismatches N*_1 _= {AA, GG, CC, UU, AC, AG, CU}; and Σ_2_, which is similar to Σ_1 _except that the mismatch set is more concise: *N*_2 _= {XX, XY}, where XX stands for one of {AA, GG, CC, UU} and XY stands for one of {AC, AG, CU}. In our alphabet, a *match*, say, AU has the same probability as UA, that is, an 'A' on either stem base paired with 'U' on the other stem. Cross-validation tests using *Maximum Likelihood Estimate *(MLE) showed that the model with alphabet Σ_1 _performed substantially better, both in terms of sensitivity and specificity (Table 2) (see *Methods *for more details on these calculations).

**Table 2:** Results for different alphabet sizes: Σ_1 _(larger alphabet) shows better accuracy than Σ_2 _(smaller alphabet)

**Alphabet**	**Sn**	**Sp**	**FDR**
Σ_1_	74.5	94.1	15.8
Σ_2_	55.0	48.5	51.0

Sn: Sensitivity; Sp: Specificity; FPR: False Positive rate; FDR: False Discovery rate. All numbers are in percentages.

It is surprising that Σ_1 _performs better than Σ_2_, because one would expect that mismatches in the stem-loop would not be characteristic of the miRNA sequence, since they do not contribute to the base pairing of the stem and thus the overall folding energy, on which other algorithms are based [23]. Furthermore, Σ_1 _alphabet has more parameters. In order to rule out that the better performance is due to parameter overfitting, we repeated training with multiple datasets of different sizes and the results remained the same (*data not shown*). In the remaining of this paper we use the Σ_1 _alphabet.

### Training algorithms: performance evaluation

**Figure 4:**
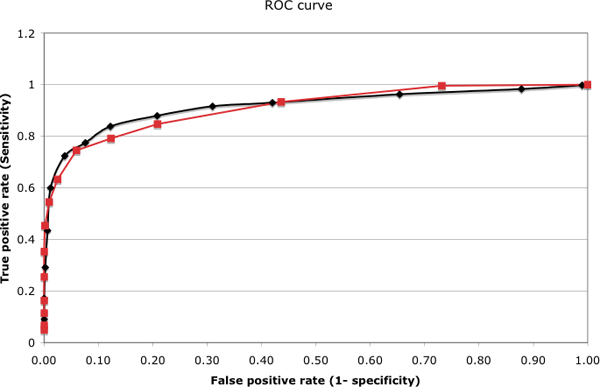
**ROC curves for Baum-Welch and MLE training on the negative model**. 10-fold cross-validation used with Baum-Welch (*black curve*) and MLE (*red curve*) for training the negative model. Positive model was trained using MLE in both cases.

We implemented and compared variations of two existing algorithms for parameter estimation: Baum-Welch and MLE. The positive model was trained using MLE since by nature the positive training data (stem-loop hairpins) can be labelled as *loop*, *extension*, *miRNA *and *pri-extension *(Figure 2b) using existing annotations. Negative data on the other hand, are obviously unlabelled, so both algorithms were compared for training with this dataset. We will call the MLE trained negative model, MLE-HHMMiR, whereas the Baum Welch trained model will be called BW-HHMMiR for this evaluation. For MLE-HHMMiR, we used length distributions from database summarization (Table 1) to perform *random labelling *of the four regions on the negative datasets. Overall, we found both methods to perform practically the same. The area under the ROC curve (Figure 4) for the MLE-HHMMiR is 0.912 whereas for BW-HHMMiR is 0.920. The ratio of the log-likelihoods output by the two models decides the fate of the test hairpin. In order to decide a threshold for this ratio, the trade-off between sensitivity and specificity was considered by calculating the *Mathews correlation coefficient *(Table 3). The highest Mathews correlation coefficient value was 0.73 for BW-HHMMiR and 0.71 for MLE-HHMMiR, corresponding to likelihood ratio thresholds of 0.71 and 0.99, respectively. BW-HHMMiR achieved an average 84% sensitivity and 88% specificity using the 0.71 ratio as thresholds. Even though, the difference between the performances of the two algorithms is not great, we choose BW-HHMMiR for further tests. This is because MLE-HHMMiR depends on *random labelling *of hairpins and thus, performance will vary according to the labelling. The drawback of the Baum-Welch method is that it might be trapped on local optima, depending on the initialization. This problem is sometimes addressed by running the algorithm multiple times with different starting points. We use a uniform distribution for this initialization but can also use background frequencies for the same by folding the entire genome in question and then performing hairpin extraction for the same. In order to account for the absence of certain base pairs or *indels *in a certain sequence while using Baum-Welch, we introduce pseudo-counts to correct for the same.

**Table 3:** Results for cross-validation using different algorithms.

**Method**	**Sn (SD)**	**Sp (SD)**	**MCC**	**FDR (SD)**
Baum-Welch	84.0 (18.6)	88.0 (6.6)	0.73	11.8 (5.6)
MLE	74.5 (13.7)	94.1 (2.7)	0.71	15.9 (8.0)

Sn: sensitivity; Sp: specificity; MCC: Mathew's correlation coefficient; FDR: False Discovery Rate. Sn, Sp and FDR report the average percent values; standard deviations are reported in parentheses.

### Testing prediction efficiency in other organisms

Next, we examined how well our model trained on human sequences could predict known miRNAs in other species. In particular, HHMMiR was tested on the following species: *M. musculus *(mammals), *G. gallus *(birds), *D. rerio *(fish), *C. elegans *(worms), *D. melanogaster *(flies), *A. thaliana *and *O. sativa*(plants). These species were chosen as representatives of their respective taxonomic groups, and because they are well studied and annotated. The results are shown in Table 4. HHMMiR is able to predict 85% of most animal precursors. Its overall sensitivity was also about 85%. What is more surprising, however, is the higher performance we observe in prediction of plant precursors, given the differences in length distributions of the miRNA stem-loops between plants and animals (Table 1). The fact that mouse miRNAs are predicted at lower rate probably reflects the larger number of hairpins registered for this species, many of which are not biochemically verified. Such discrepancies have been observed in other studies as well, although at a lesser extent (*e.g*., [25]). The specificity over the mouse data is very high (84%) and remains surprisingly high in the two invertebrate species (~75%) (*data not shown*).

**Table 4:** Results of tests on other species.

**Organism**	**Total hairpins**	**% correctly predicted**
*M. musculus*	422	74.7
*G. gallus*	147	89.1
*D. rerio*	334	88.3
*C. elegans*	131	85.5
*D. melanogaster*	143	93.0
*A. thaliana*	114	97.4
*O. sativa*	188	85.7

**Total**	**1479**	**85.1**

### Comparison with other approaches

As described earlier, there are very few machine learning methods that do not require evolutionary information to predict miRNAs. To our knowledge, the only other probabilistic model is a motif finding method for mature miRNA region prediction [32]. An SVM-based approach has been proposed [25] that parses the *mfe *structure in "triplets": structural information about the pairing states of every three nucleotides, represented using dot-bracket notation. This method showed an accuracy of ~90% using the data available in the registry at the time. We used the same training and test sets used by the "triplet SVM" to train and test our model, HHMMiR, and we found it to perform better in almost all datasets (Table 5). The only exceptions are the mouse (but not rat) and *A. thaliana *(but not rice). Also, their model was able to predict all of the then five known miRNAs from Epstein-Barr virus, whereas HHMMiR predicted four. Overall, HHMMiR exhibits sensitivity of 93.2% and specificity of 89% in these datasets. If we limit the comparison of the two methods in one representative species from each taxon (*M. musculus*, *G. gallus*, *D. rerio*, *C. elegans*, *D. melanogaster*, *A. thaliana*, *Epstein Barr virus*) in order to minimize the statistical dependence of the data, the difference in the performance becomes statistically significant at the 5% level (*p*-value = 0.03, Wilcoxon paired test on the predicted number of genes).

**Table 5:** Results for comparison between two precursor prediction methods.

Test Set	Total hairpins	Triplet SVM (%)	HHMMiR (%)
**Positive Sets**			
New human hairpins in registry at the time.	39	92.3	97.4
*M. musculus*	36	94.4	88.9
*R. norvegicus*	25	80.0	84.0
*G. gallus*	13	84.6	100
*D. rerio*	6	66.7	100
*C. elegans*	110	86.4	90.9
*C. briggsae*	73	95.9	95.9
*D. melanogaster*	71	91.6	95.8
*D. pseudoobscura*	71	90.1	98.6
*A. thaliana*	75	92.0	97.3
*O. sativa*	96	94.8	86.5
*Epstein Barr virus*	5	100	80.0
**TOTAL**	**620**	**91**	**93.2**

**Negative Sets**			
Folded genome hairpins from Chromosome 19	2444	89	88.6
Negative hairpin Set	1000	88.1	89.4
**TOTAL**	**3444**	**88.7**	**88.8**

The percentages represent the ratio of hairpins correctly predicted.

## Discussion

MiRNA genes constitute one of the most conserved mechanisms for gene regulation across all animal and plant species. The characteristics of the precursor miRNA stem-loops are well conserved in both vertebrate and invertebrate animals and fairly conserved between animals and plants. As seen in Table 1, plant hairpins tend to be generally longer than those in animals, while vertebrates have shorter precursors than invertebrates. Although, the "extension" and "pri-extension" regions may vary in length between animals and plants (much longer in plants), the lengths of the "miRNA" and "loop" regions are more similar. Thus, even across evolutionary time, the basic characteristics of miRNAs have not changed dramatically.

We designed a template for a typical precursor miRNA stem-loop and we built an HHMM based on it. HHMMiR was able to attain an average sensitivity of 84% and specificity of 88% on 10-fold cross validation of human data. We trained HHMMiR on human sequences and the resulting model was able to successfully identify a large percentage of not only mouse, but also invertebrate, plant and virus miRNAs (Table 4). This is an encouraging result showing that HHMMiR may be very useful in predicting miRNA genes across long evolutionary distances without the requirement for evolutionary conservation of sequences. This would be very beneficial for identification of miRNA hairpins in organisms that do not have closely related species sequenced, such as *Strongylocentrotus purpuratus *(sea urchin) and *Ornithorhynchus anatinus *(platypus) [33].

This is the first time a hierarchical probabilistic model has been used for classification and identification of miRNA hairpins. Probabilistic learning was previously applied by Nam *et al. *[32] for identifying the miRNA pattern/motif in hairpins. The advantage of the hierarchy used by our HHMMiR is that it parses each hairpin into four distinct regions and processes each of them separately. This represents better the biological role of each region, which is reflected in the distinct length distributions and neighbourhood base-pairing characteristic of that region. Furthermore, the underlying HHMM provides an intuitive modelling of these regions. We compared two modifications of the MLE and Baum-Welch algorithms for modelling the negative datasets, and we found them to perform similarly. Baum-Welch was selected for this study, since it does not require (random) labelling of the negative set.

The drawback of HHMMiR is that it depends on the *mfe *structure the *RNAfold *program returns [31]. In the future, we will test more folding algorithms or use the probability distribution of a number of top scoring folding energy structures returned by this package.

## Conclusion

The success of our approach shows that the conservation of the miRNA mechanism may be at a much deeper level than expected. Further developments of the HHMMiR algorithm include the extension of the precursor template model (Figure 3) to be able to predict pri-miRNA genes with multiple stem-loops. Another extension would be to train a model to decode all HHMMiR predicted hairpins to identify the miRNA genes in them. Finally, it will be interesting to extend our method to include evolutionary information, which will allow us to assess the significance of conservation in predicting miRNA genes.

## Methods

### Data collection and processing

#### MiRNA dataset

MiRNA genes were obtained from the *microRNA registry*, version 10.1 (December 2007) [34], which contains 3265 miRNAs from animals and 870 from plants. For training HHMMiR, we used the residual 525 human hairpins, after filtering out precursor genes with multiple loops. Each gene was folded with the *RNAfold *program, which is part of the Vienna package [31], using the default parameters to obtain the secondary structure with minimum fold energy. The negative set consists of coding regions and random genomic segments from the human genome that were obtained using the UCSC genome browser [35]. These regions were folded and processed as described below.

#### Hairpin processing

**Figure 5:**
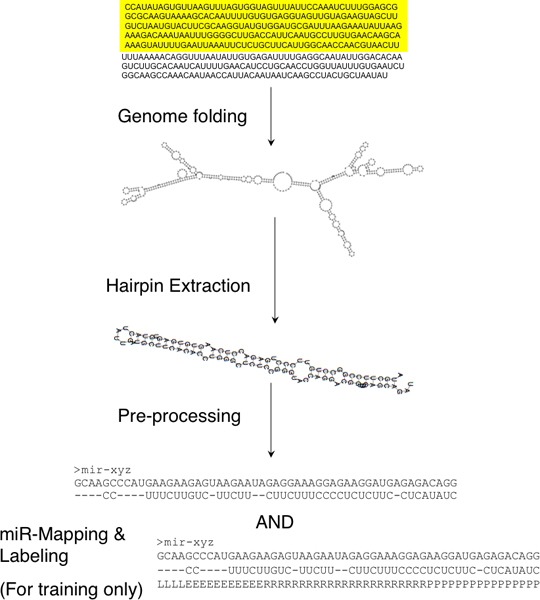
**Data flow for hairpin extraction from the genome**. The genome is first folded using windows of 500 nts with 150 nts overlap between consecutive windows. Hairpins are then extracted from the folded windows using the parameters described in the text. Hairpins are pre-processed into a suitable format for training/testing using the states shown in Figure 3 (L: Loop; E: Extension; R: miRNA; P: pri-miRNA extension). For the purpose of testing, the folded sequence is pre-processed into 2 lines of input representing the 2 stems of the hairpin. An example is given in Figure 2b.

Genomic sequences were folded in windows of 1 Kb, 500 nts and 300 nts with an overlap of 150 nts between consecutive windows. Nodes from the TeraGrid project [36] were used for this purpose. We tested the various window sizes on the relatively small *C. elegans *genome and discovered that 500 nts windows cover most known miRNA hairpins. Windows of 300 nts exhibited high degree of redundancy without adding more hairpins to those of the 500 nts windows, while 1 kb windows missed a higher percentage of known miRNAs (*data not shown*). For this study, we used hairpins extracted from windows of 500 nts. We were able to recover ~92% of the known miRNAs from *C. elegans *in this way. The remaining 8% may have been accounted for by existence of multiple loops or specificity of the parameters used. The hairpins were extracted from these folded windows using the following parameters: each hairpin has at least 10 base pairs, has a maximum length of 20 bases for the loop, and a minimum length of 50 nucleotides. The data flow of this process is presented in Figure 5.

After the hairpins are extracted, we process them to an input format representing the hairpin's secondary structure (Figure 5 and Figure 2) to be compatible with the HHMM shown in Figure 3. The labelling is done only for training data. For the purpose of labelling, the miRNA is first mapped to the folded hairpin (on either or both arms), and then the region representing the miRNA is labelled as the duplex miRNA (R) region. Our method does not consider the 3' overhangs generated during Dicer processing. The main bulge is labelled as the loop (L), whereas the remaining region between loop and miRNA is represented as the extension (E). The rest of the hairpin beyond the miRNA is labelled as pri-extension (P). A detailed description of these regions is given in the *Results *section.

### Parameter estimation and testing

#### Parameter estimation

Two separate HHMM models are trained, one on positive data set (miRNAs and their corresponding hairpins) and the other on negative data set (hairpins, randomly chosen from the coding parts of the genome). The hairpins are pre-processed and labelled (if needed) before parameter estimation. Baum-Welch requires no labelling, but for MLE, we applied random labelling, as described above (Figure 2a).

The *alphabet *is denoted by Σ = {σ_*i*_} and the observed finite string is denoted by ***O ***= *o*_1_*o*_2 _... *o*_*N *_such that *o*_*i *_∈ Σ. The *i*^*th *^state at hierarchical level *d *is denoted as $q_{i}^{d}$ (denoted as *q*^*d *^in absence of ambiguity). The highest level of hierarchy (that of the root) is 1 while the lowest (that of the production states) is *D *(in our case, *D *= 3). The number of substates of each $q_{i}^{d}$ (*d *∈ {1, 2, ... *D-1*} is |$q_{i}^{d}$|. The parameter set of an HHMM is denoted by:

$$\lambda ={\left\{\lambda^{q^{d}}\right\}}_{d\in \left\{1,...,D\right\}}=\left\{\begin{array}{l} {\left\{A\left(q^{d}\right)\right\}}_{d\in \left\{1,...,D\right\}},\\ {\left\{\prod \left(q^{d}\right)\right\}}_{d\in \left\{1,...,D\right\}},\\ \left\{E\left(q^{D}\right)\right\} \end{array}\right\}$$

$A\left(q^{d}\right)=\left(a_{jk}^{q^{d}}\right)=P\left(q_{k}^{d+1}|q_{j}^{d+1}\right)$ denoted by ${\left\{A\left(q^{d}\right)\right\}}_{d\in \left\{1,...,D\right\}}$ is the state *transition matrix *of each internal substate, with $a_{jk}^{q^{d}}=P\left(q_{j}^{d+1}|q_{i}^{d+1}\right)$ representing the probability that the *j*^*th *^substate of *q*^*d *^will transition to the *k*^*th *^substate of *q*^*d*^. Each internal state *q*^*d *^has also an *initial distribution vector *$\prod \left(q^{d}\right)=\left\{\pi \left(q_{j}^{d+1}|q^{d}\right)\right\}=\left\{P\left(q_{j}^{d+1}|q^{d}\right)\right\}$ denoted by ${\left\{\prod \left(q^{d}\right)\right\}}_{d\in \left\{1,...,D\right\}}$, where $\pi \left(q_{j}^{d+1}|q^{d}\right)$ is the probability that *q*^*d *^will make a vertical transition to its *j*^*th *^substate at level *d+*1, thus, activating it. The production states *q*^*D *^will have *emission probability vector *or the *output distribution vector *$E\left(q^{D},q^{D-1}\right)=\left\{e\left(\sigma_{l}|q^{D},q^{D-1}\right)\right\}=\left\{P\left(\sigma_{l}|q^{D},q^{D-1}\right)\right\}$ denoted by {*E*(*q*^*D*^)} where *e*(σ_*l*_|*q*^*D*^, *q*^*D*-1^) is the probability that production state *q*^*D *^will emit symbol *σ*_*l *_∈ Σ.

Now we will define the various probabilities that are required to be calculated for parameter estimation.

(*i*) $\alpha \left(t,t+k,q_{i}^{d+1},q^{d}\right)=P(o_{t}\cdot \cdot \cdot o_{t+k},q_{i}^{d+1}$ finished at $o_{t+k}|q^{d}$ started at *o*_*t*_) is the *forward probability *of emitting the substring *o*_*t *_... *o*_*t*+*k *_of the observation sequence by the parent state *q*^*d *^such that it was entered at *o*_*t *_and the subsequence ended at substate $q_{i}^{d+1}$ and thus, it was the last state activated.

(*ii*) $\chi \left(t,q_{i}^{d+1},q^{d}\right)$ is the probability of making a vertical transition from parent *q*^*d *^to $q_{i}^{d+1}$ just before the emission of *o*_*t*_.

(*iii*) $\xi \left(t,q_{i}^{d+1},q_{j}^{d+1},q^{d}\right)=P(o_{1}\cdot \cdot \cdot o_{t},q_{i}^{d+1}\to q_{j}^{d+1},o_{t+1}\cdot \cdot \cdot o_{N}|\lambda )$ is the probability of making a horizontal transition from $q_{i}^{d+1}$ to $q_{j}^{d+1}$ where both are substates of *q*^*d *^after the emission of *o*_*t *_and before the emission of *o*_*t*+1_.

(*iv*) $\gamma_{in}\left(t,q_{i}^{d+1},q^{d}\right)=\sum_{k=1}^{\left|q^{d}\right|}\xi \left(t-1,q_{k}^{d+1},q_{i}^{d+1},q^{d}\right)$ is the probability of performing a horizontal transition to $q_{i}^{d+1}$ which is substate of *q*^*d *^before *o*_*t *_is emitted. Further details on the algorithms are given in [27] and in Additional file 2.

The parameters are estimated as follows:

$$\begin{array}{l} \hat{\pi }\left(q_{i}^{2}|q^{1}\right)=\chi \left(t,q_{i}^{2},q^{1}\right)\\ \begin{array}{cc} \hat{\pi }\left(q_{i}^{d+1}|q^{d}\right)=\frac{\sum_{t=1}^{T}\chi \left(t,q_{i}^{d+1},q^{d}\right)}{\sum_{i=1}^{\left|q^{d}\right|}\sum_{t=1}^{T}\chi \left(t,q_{i}^{d+1},q^{d}\right)} & \left(1<d<D-1\right) \end{array}\\ {\hat{a}}_{jk}^{q^{d}}=\frac{\sum_{t=1}^{T}\xi \left(t,q_{i}^{d+1},q_{j}^{d+1},q^{d}\right)}{\sum_{k=1}^{\left|q^{d}\right|}\sum_{t=1}^{T}\xi \left(t,q_{i}^{d+1},q_{k}^{d+1},q^{d}\right)}\\ \begin{array}{c} \hat{e}\left(\sigma_{l}|q^{D},q^{D-1}\right)=(\sum_{o_{t}=\sigma_{l}}\chi \left(t,q_{i}^{D},q^{D-1}\right)\\ +\sum_{t>1,o_{t}=\sigma_{l}}\gamma_{in}\left(t,q_{i}^{D},q^{D-1}\right))/(\sum_{t=1}^{T}\chi \left(t,q_{i}^{D},q^{D-1}\right)\\ +\sum_{t=2}^{T}\gamma_{in}\left(t,q_{i}^{D},q^{D-1}\right)) \end{array} \end{array}$$

#### Testing

As described above, classification of test hairpins depends on the ratio of the log-likelihoods generated by the positive and negative models. A threshold was decided for this ratio using the ROC curves shown in Figure 4. For each hairpin, the probability that a certain model emitted the hairpin is given by:

$$P\left(O|\lambda \right)=\sum_{i=1}^{\left|q^{1}\right|}\alpha \left(1,T,q_{i}^{2},q^{1}\right)$$

#### Measures of accuracy

The different terms and measures used to calculate the efficiency of HHMMiR are listed in the Table 6.

**Table 6:** Measures for accuracy calculation.

**Measure**	**Calculation**
Sensitivity (Sn)	Sn=TPTP+FN
Specificity (Sp)	Sp=TNTN+FP
False Discovery Rate (FDR)	FDR=FPTP+FP
Matthew's Correlation Coefficient (MCC)	$MCC=\left(TP\cdot TN-FP\cdot FN\right)/\left(\sqrt{\left(TP+FP)\cdot (TP+FN)\cdot (TN+FP)\cdot (TN+FN\right)}\right)$

TP: *True Positives*; TN: *True Negatives*; FP: *False Positives*; FN: *False Negatives*.

## List of abbreviations used

HHMM: hierarchical hidden Markov model; mfe: minimum fold energy; miRNA: microRNA; MLE: maximum likelihood estimate.

## Competing interests

The authors declare that they have no competing interests.

## Authors' contributions

PVB and SK designed the study, analyzed the results and wrote the paper. SK implemented the HHMM. VH supervised the data analysis and contributed to the writing of the paper.

## Supplementary Material

Additional File 1 This file contains the results of summarization of the *microRNA registry *(version 10.1, December 2007) [34] hairpin characteristics for each species. Click here for file

Additional File 2 This file contains a more detailed description of the algorithms used for parameter estimation and classification using HHMMs. Click here for file
