# Supplementary material for: HHMMiR: efficient de novo prediction of microRNAs using hierarchical hidden Markov models
Source: BMC Bioinformatics. 2009 Jan 30;10(Suppl 1):S35. doi: 10.1186/1471-2105-10-S1-S35 (PMC2648761; doi:10.1186/1471-2105-10-S1-S35)
Supplement: Additional File 2 — This file contains a more detailed description of the algorithms used for parameter estimation and classification using HHMMs. [file 1471-2105-10-S1-S35-S2.pdf]

Supplementary Information for:

"HHMMiR: Efficient de novo Prediction of MicroRNAs using Hierarchical Hidden Markov Models",

by

Sabah Kadri, Veronica Hinman, Panayiotis V. Benos

APBC 2009 and *BMC Bioinformatics* 2009

Symbolism.

Finite alphabet: $\sum$

Observed string: $O = o_1 o_2 \ldots o_N$ such that $o_i \in \sum$

Highest level of hierarchy (root): 1

Lowest level of hierarchy (leaves): D

Depth of hierarchy: $d \in \{1,\ldots,D\}$

i^{th} state at hierarchical level d: q_i^d

Number of substates of q_i^d: $\left|q_i^d\right|$

Parameters of HHMM:

$$\lambda = \left\{\lambda^{q^d}\right\}_{d\in\{1,\ldots,D\}} = \left\{\begin{array}{l} \left\{A\left(q^d\right)\right\}_{d\in\{1,\ldots,D\}}, \\ \left\{\prod\left(q^d\right)\right\}_{d\in\{1,\ldots,D\}}, \\ \left\{E\left(q^D\right)\right\} \end{array}\right\}$$

1. **Substate Transition Matrix:**

$$\left\{A\left(q^d\right)\right\}_{d\in\{1,\ldots,D\}} \text{ such that } A\left(q^d\right) = \left(a_{jk}^{q^d}\right) = P\left(q_k^{d+1} \middle| q_j^{d+1}\right)$$

$a_{jk}^{q^d}$ is the probability that the jth substate of q^d will transition to its kth substate.

2. **Initial Substate distribution:**

$\left\{\prod\left(q^d\right)\right\}_{d\in\{1,\ldots,D\}}$ such that $\prod\left(q^d\right)=\left\{\pi\left(q_j^{d+1}\middle|q^d\right)\right\}=\left\{P\left(q_j^{d+1}\middle|q^d\right)\right\}$

$\pi\left(q_j^{d+1}\middle|q^d\right)$ is the probability that q^d will make a vertical transition to its jth substate at level $d+1$.

3. **Output probability distribution:**

$\left\{E\left(q^D\right)\right\}$ such that $E\left(q^D, q^{D-1}\right)=\left\{e\left(\sigma_l\middle|q^D, q^{D-1}\right)\right\}=\left\{P\left(\sigma_l\middle|q^D, q^{D-1}\right)\right\}$

$e\left(\sigma_l\middle|q^D, q^{D-1}\right)$ is the probability that production state q^D will emit symbol $\sigma_l \in \sum$.

Modified Baum Welch algorithm

Calculate the following probabilities:

1. **Forward Probabilities**

$\alpha\left(t, t+k, q_i^{d+1}, q^d\right)=P(o_t \cdots o_{t+k}, q_i^{d+1}$ finished at $o_{t+k}\,|\,q^d$ started at $o_t)$

Initialization:

Production states:

$$\alpha\left(t, t, q_i^D, q^{D-1}\right)=\pi\left(q_i^D\middle|q^{D-1}\right)e\left(o_t\middle|q_i^D, q^{D-1}\right)$$

Internal States:

$$\alpha\left(t,t,q_i^d,q^{d-1}\right)=\pi\left(q_i^d\middle|q^{d-1}\right)\left[\sum_{j=1}^{\left|q_i^d\right|}\alpha\left(t,t,q_j^{d+1},q_i^d\right)\cdot a_{j\,end}^{q_i^d}\right]$$

Iteration:

Production states:

$$\alpha\left(t,t+k,q_i^D,q^{D-1}\right)=\left[\sum_{j=1}^{\left|q^{D-1}\right|}\alpha\left(t,t+k-1,q_j^D,q^{D-1}\right)\right]e\left(o_{t+k}\middle|q_i^D,q^{D-1}\right)$$

Internal States:

$$\alpha\left(t,t+k,q_i^d,q^{d-1}\right)=\sum_{l=0}^{k-1}\left[\sum_{j=1}^{\left|q^{d-1}\right|}\alpha\left(t,t+l,q_j^d,q^{d-1}\right)\cdot a_{ji}^{q^{d-1}}\right]\cdot$$
$$\left[\sum_{s=1}^{\left|q_i^d\right|}\alpha\left(t+l+1,t+k,q_s^{d+1},q_i^d\right)\cdot a_{s\,end}^{q_i^d}\right]$$
$$+\pi\left(q_i^d\middle|q^{d-1}\right)\left[\sum_{j=1}^{\left|q_i^d\right|}\alpha\left(t,t+k,q_j^{d+1},q_i^d\right)\cdot a_{j\,end}^{q_i^d}\right]$$

2. **Backward Probabilities**

$$\beta\left(t,t+k,q_i^d,q^{d-1}\right)=P\left(o_t\cdots o_{t+k}\middle|q_i^d\text{ started at }o_t,\ q^{d-1}\text{ finished at }o_{t+k}\right)$$

Initialization:

Production states:

$$\beta\left(t,t,q_i^D,q^{D-1}\right)=e\left(o_t\middle|q_i^D,q^{D-1}\right)\cdot a_{i\,end}^{q^{D-1}}$$

Internal States:

$$\beta\left(t,t,q_i^d,q^{d-1}\right)=\left[\sum_{j=1}^{\left|q_i^d\right|}\pi\left(q_j^{d+1}\middle|q_i^d\right)\cdot\beta\left(t,t,q_j^{d+1},q_i^d\right)\right]a_{i\,end}^{q^{d-1}}$$

Iteration:

Production states:

$$\beta\left(t, t+k, q_i^D, q^{D-1}\right) = e\left(o_t \middle| q_i^D, q^{D-1}\right)\left[\sum_{j \neq end}^{\left|q^{D-1}\right|} a_{ij}^{q^{D-1}} \cdot \beta\left(t+1, t+k, q_j^D, q^{D-1}\right)\right]$$

Internal States:

$$\beta\left(t, t+k, q_i^d, q^{d-1}\right) = \sum_{l=0}^{k-1}\left[\sum_{j=1}^{\left|q_i^d\right|} \pi\left(q_j^{d+1} \middle| q_i^d\right)\beta\left(t, t+l, q_j^{d+1}, q_i^d\right)\right] \cdot$$

$$\left[\sum_{s=1}^{\left|q^{d-1}\right|} a_{ij}^{q^{d-1}} \cdot \beta\left(t+l+1, t+k, q_s^d, q^{d-1}\right)\right]$$

$$+\left[\sum_{j=1}^{\left|q_i^d\right|} \pi\left(q_j^{d+1} \middle| q_i^d\right) \cdot \beta\left(t, t+k, q_j^{d+1}, q_i^d\right) \cdot a_{i\,end}^{q^{d-1}}\right]$$

3. **Auxiliary variables:**

A. $\eta_{in}\left(t, q_i^d, q^{d-1}\right) = P\left(o_1 \cdots o_{t-1}, q_i^d \text{ started at } o_t \middle| \lambda\right)$

<u>Initialization:</u>

$$\eta_{in}\left(1, q_i^2, q^1\right) = \pi\left(q_i^2 \middle| q^1\right)$$

$$\eta_{in}\left(1, q_i^d, q_j^{d-1}\right) = \eta_{in}\left(1, q_j^{d-1}, q^{d-2}\right) \cdot \pi\left(q_i^d \middle| q_j^{d-1}\right)$$

<u>Iteration:</u>

For 1<t

$$\eta_{in}\left(t, q_i^2, q^1\right) = \sum_{j=1}^{\left|q^1\right|} \alpha\left(1, t-1, q_j^2, q^1\right) a_{ji}^{q^1}$$

$$\eta_{in}\left(t, q_i^d, q_j^{d-1}\right) = \sum_{s=1}^{t-1} \eta_{in}\left(s, q_j^{d-1}, q^{d-2}\right)\left[\sum_{l=1}^{\left|q_j^{d-1}\right|} \alpha\left(s, t-1, q_l^d, q_j^{d-1}\right) a_{li}^{q_j^{d-1}}\right]$$

$$+\eta_{in}\left(t, q_j^{d-1}, q^{d-2}\right) \cdot \pi\left(q_i^d \middle| q_j^{d-1}\right)$$

B. $\eta_{out}\left(t, q_i^d, q^{d-1}\right) = P\left(q_i^d \text{ finished at } o_t, o_{1t+1} \cdots o_N \middle| \lambda\right)$

Initialization:

For $t<N$

$$\eta_{out}\left(t,q_i^2,q^1\right)=\sum_{j=1}^{\left|q^1\right|}a_{ij}^{q^1}\cdot\beta\left(t+1,N,q_j^2,q^1\right)$$

Iteration:

For $t<N$

$$\eta_{in}\left(t,q_i^d,q_j^{d-1}\right)=\sum_{k=t+1}^{N}\left[\sum_{l=1}^{\left|q_j^{d-1}\right|}a_{il}^{q_j^{d-1}}\beta\left(t+1,N,q_l^d,q_j^{d-1}\right)\right]\eta_{out}\left(k,q_j^{d-1},q^{d-2}\right)$$
$$+a_{i\,end}^{q_j^{d-1}}\cdot\eta_{out}\left(t,q_j^{d-1},q^{d-2}\right)$$

$$\eta_{out}\left(N,q_i^d,q_j^{d-1}\right)=a_{j\,end}^{q_j^{d-1}}\cdot\eta_{out}\left(N,q_j^{d-1},q^{d-2}\right)$$

4. **Horizontal Transition Probabilities**

$$\xi\left(t,q_i^{d+1},q_j^{d+1},q^d\right)=P(o_1\cdots o_t,q_i^{d+1}\rightarrow q_j^{d+1},o_{t+1}\cdots o_N\left|\lambda\right.)$$

Estimation:

For $t<N$

$$\xi\left(t,q_i^2,q_j^2,q^1\right)=\frac{\alpha\left(1,t,q_i^2,q^1\right)\cdot a_{ij}^{q^1}\cdot\beta\left(t+1,N,q_j^2,q^1\right)}{P\left(O\middle|\lambda\right)}$$

$$\xi\left(N,q_i^2,q_j^2,q^1\right)=\frac{\alpha\left(1,N,q_i^2,q^1\right)\cdot a_{ij}^{q^1}}{P\left(O\middle|\lambda\right)}$$

For $t<N$

$$\xi\left(t,q_i^d,q_j^d,q_l^{d-1}\right)=\frac{1}{P\left(O\middle|\lambda\right)}\left[\sum_{s=1}^{t}\eta_{in}\left(s,q_l^{d-1},q^{d-2}\right)\cdot\alpha\left(s,t,q_i^d,q_l^{d-1}\right)\right]a_{ij}^{q_l^{d-1}}$$
$$+\left[\sum_{k=t+1}^{N}\beta\left(t+1,k\middle|q_j^d,q_l^{d-1}\right)\cdot\eta_{out}\left(k,q_l^{d-1},q^{d-2}\right)\right]$$

$$\xi\left(t, q_i^d, q_{end}^d, q_j^{d-1}\right) = \frac{1}{P\left(O\middle|\lambda\right)}\left[\sum_{s=1}^{t}\eta_{in}\left(s, q_j^{d-1}, q^{d-2}\right)\cdot\alpha\left(s, t, q_i^d, q_j^{d-1}\right)\right]$$

$$a_{i\,end}^{q_j^{d-1}}\cdot\eta_{out}\left(t, q_j^{d-1}, q^{d-2}\right)$$

5. **Vertical Transition Probabilities**

$$\chi\left(t, q_i^d, q^{d-1}\right) = P\left(q_i^d \text{ started at } t\middle|\lambda, O\right)$$

$$= P(o_1\cdots o_{t-1}, \underset{q_i^d}{\overset{q^{d-1}}{\downarrow}}, o_t\cdots o_N\left|\lambda, O\right.)$$

Initiation:

$$\chi\left(1, q_i^2, q^1\right) = \frac{\pi\left(q_i^2\middle|q^1\right)\cdot\beta\left(1, N, q_i^2, q^1\right)}{P\left(O\middle|\lambda\right)}$$

Iteration:

For *2<d*

$$\chi\left(t, q_i^d, q_j^{d-1}\right) = \frac{\eta_{in}\left(t, q_j^{d-1}, q^{d-2}\right)\cdot\pi\left(q_i^d\middle|q_j^{d-1}\right)}{P\left(O\middle|\lambda\right)}$$

$$\left[\sum_{k=t}^{N}\beta\left(t, k, q_i^d, q_j^{d-1}\right)\cdot\eta_{out}\left(k, q_j^{d-1}, q^{d-2}\right)\right]$$

Parameter Estimation

1. $\gamma_{in}\left(t, q_i^{d+1}, q^d\right)$ is the probability of performing a horizontal transition to q_i^{d+1} which is substate of q^d before o_t is emitted

$$\gamma_{in}\left(t, q_i^{d+1}, q^d\right) = \sum_{k=1}^{\left|q^d\right|}\xi\left(t-1, q_k^{d+1}, q_i^{d+1}, q^d\right)$$

2. $\gamma_{out}\left(t, q_i^{d+1}, q^d\right)$ is the probability of performing a horizontal transition from q_i^{d+1} which is substate of q^d to any of the other substates of q^d after o_t is emitted

$$\gamma_{out}\left(t, q_i^{d+1}, q^d\right) = \sum_{k=1}^{\left|q^d\right|} \xi\left(t, q_i^{d+1}, q_k^{d+1}, q^d\right)$$

Thus,

$$\hat{\pi}\left(q_i^2 \middle| q^1\right) = \chi\left(t, q_i^2, q^1\right)$$

$$\hat{\pi}\left(q_i^{d+1} \middle| q^d\right) = \frac{\sum_{t=1}^{T} \chi\left(t, q_i^{d+1}, q^d\right)}{\sum_{i=1}^{\left|q^d\right|} \sum_{t=1}^{T} \chi\left(t, q_i^{d+1}, q^d\right)} \quad \left(1 < d < D-1\right)$$

$$\hat{a}_{jk}^{q^d} = \frac{\sum_{t=1}^{N} \xi\left(t, q_i^{d+1}, q_j^{d+1}, q^d\right)}{\sum_{k=1}^{\left|q^d\right|} \sum_{t=1}^{N} \xi\left(t, q_i^{d+1}, q_k^{d+1}, q^d\right)} = \frac{\sum_{t=1}^{N} \xi\left(t, q_i^{d+1}, q_j^{d+1}, q^d\right)}{\sum_{t=1}^{N} \gamma_{out}\left(t, q_i^{d+1}, q^d\right)}$$

$$\hat{e}\left(\sigma_l \middle| q^D, q^{D-1}\right) = \Big(\sum_{o_t = \sigma_l} \chi\left(t, q_i^D, q^{D-1}\right)$$

$$+ \sum_{t>1, o_t = \sigma_l} \gamma_{in}\left(t, q_i^D, q^{D-1}\right) \Big) / \Big(\sum_{t=1}^{T} \chi\left(t, q_i^D, q^{D-1}\right)$$

$$+ \sum_{t=2}^{T} \gamma_{in}\left(t, q_i^D, q^{D-1}\right) \Big)$$
